# Aqueous double-layer paint of low thickness for sub-ambient radiative cooling

**DOI:** 10.1515/nanoph-2023-0664

**Published:** 2024-01-18

**Authors:** Benjamin Dopphoopha, Keqiao Li, Chongjia Lin, Baoling Huang

**Affiliations:** The Department of Mechanical and Aersopace Engineering, The Hong Kong University of Science and Technology, Clear Water Bay, Kowloon, Hong Kong, China; The Department of Mechanical and Aersopace Engineering, Foshan Research Institute for Smart Manufacturing, The Hong Kong University of Science and Technology, Clear Water Bay, Kowloon, Hong Kong, China; HKUST Shenzhen-Hong Kong Collaborative Innovation Research Institute, Futian, Shenzhen 518000, China; Thrust of Sustainable Energy and Environment, The Hong Kong University of Science and Technology, Guangzhou, China

**Keywords:** multilayer, radiative cooling, Monte Carlo simulation, water-based acrylic paint

## Abstract

Radiative cooling may serve as a promising option to reduce energy consumption for space cooling. Radiative cooling paints provide a cost-effective and scalable solution for diverse applications and attract great attention, but the state-of-art cooling paints generally use non-eco-friendly organic solvents and need large thicknesses (>400 μm) to realize high performance, which leads to high cost and environmental issues in implementation. This work aims to address these challenges by developing eco-friendly aqueous paints with low thickness (below 150 μm) by adopting a double-layer design based on a complementary spectrum strategy. The structure consists of a wide bandgap top layer to scatter short-wavelength light and a bottom layer with high reflectance to visible and near-infrared (NIR) irradiation. Effects of different design factors are studied using numerical simulation and experiments to attain the optimal design. The resulting Y_2_O_3_–ZnO paints show a strong reflectance of 95.4 % and a high atmospheric window emissivity of 0.93 at a low thickness of 150 μm. Field tests in the subtropic humid climate of Hong Kong demonstrated sub-ambient cooling of 2 °C at noon and 4 °C at night without shielding convection. The paints also show high robustness and excellent resistance to water and UV light attacks, rendering them promising for large-scale applications.

## 1 Introduction

Carbon emission reduction has become a worldwide consensus for future sustainable development. Current compressor-based active air conditioning relies on electricity for operation and may contribute around 30 % of total electricity consumption [1], [2], rendering it a major carbon emission source in urban regimes [3]. Thus, cooling technologies [4], [5], [6] that require less to net-zero energy consumption have been actively sought out and investigated in recent decades. Radiative cooling is an emerging eco-friendly cooling approach requiring zero energy input. It achieves cooling by emitting thermal radiation in the atmospheric windows (normally within the 8–13 µm wavelength range) [7], [8] to the higher sky and preventing further heat generation by reflecting solar irradiation. Various radiative cooling coatings have been developed over the recent years and succeeded in cooling to sub-ambient temperatures [9]–[23], such as inorganic particles dispersion [9], [10], porous polymers [11], [12], [13], micro-structures [14], and nano-fabricated layered structures [15], [16], [17], [18]. Among the different cooling coatings, radiative cooling paints are widely considered one of the most cost-effective and scalable solutions, which can be potentially applied in diverse scenarios including buildings, textiles, chemical storage, and other outdoor facilities, owing to their merits such as low cost, high throughput production, and easy implementation. Various cooling paints that used calcium phosphate [19], silicon oxide beads [20], and other fillers [9], [21]–[28] have shown high solar reflectivity and excellent sub-ambient cooling performance. While they show that radiative cooling by paint is possible, certain issues arise. First, the paints reported in the literature generally need a large thickness ranging from 300 to 500 µm to achieve high solar reflectance [19], [24], [29]. This can lead to significant weight bearing on the substrate of interest, high cost to paint and maintain, and over-insulation for heat dissipation from the buildings. Meanwhile, most of the reported cooling paints are based on volatile and flammable organic solvents such as toluene and dimethylformamide (DMF). They may raise concerns about operation/storage safety, odor, and health hazards including volatile organic compounds (VOC) emission. Non-toxic and eco-friendly aqueous cooling paints of high performance and low mass loading are thus highly desirable for practical applications [30], [31], [32].

This work proposes a double-layer structure that exploits a complementary spectrum strategy to reduce the thickness of a painted coating while maintaining good radiative cooling performance. This structure consists of a top layer that strongly reflects UV radiation and a bottom layer that mainly scatters the visible and NIR light, thus realizing near-perfect full solar spectrum reflection with a small thickness down to 150 μm. To optimize the design, Monte Carlo simulations are conducted to determine the key parameters that influence the scatterings in different wavelength regimes for minimizing the coating thickness. An aqueous Y_2_O_3_–ZnO double-layer paint is then developed, which shows a high solar reflectance of 95.4 % and a thermal emissivity of 0.93 with a total thickness of 150 μm. Field tests of the developed ultrathin paint were conducted in summer in Hong Kong, a coastal city with a hot humid sub-tropic climate, demonstrating a sub-ambient cooling of 2 °C at noon and 4 °C at night without convection cover. The paint also shows good hydrophobicity, strong adhesion, and excellent durability such as high UV and water resistance, which are critical for practical large-scale applications. We further demonstrated that this design strategy can be extended for the development of other thin radiative cooling paints such as Y_2_O_3_–TiO_2_ paints.

## 2 Design and modelling

**Figure 1:**
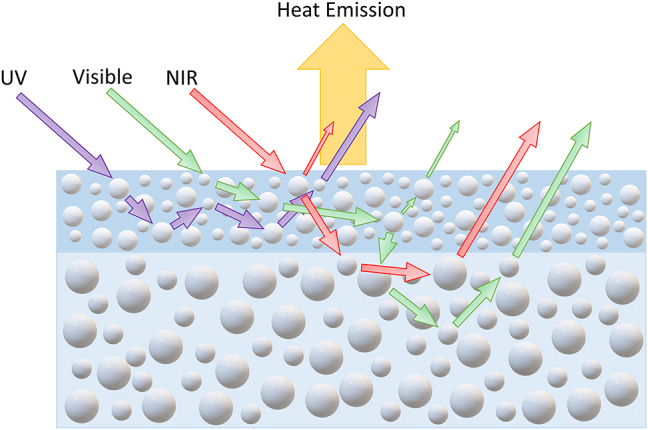
Schematic of double-layer paint structure and its working principle.

Cooling paints reported in the literature are often a single-layer design, which consists of white pigments (or fillers) and other additives embedded in a resin matrix. These paints rely on the multiple scatterings by the microparticles or pores in the matrix, which can hardly be optimized to produce superior scattering efficiency throughout the entire solar spectrum and thus generally need a large thickness (∼400–500 μm) to achieve a high solar reflectance. Therefore, the thickness of paint coating can be reduced by adopting a multilayer design, in which different layers may be optimized for targeted irradiation regimes. On the other hand, the number of layers should not be too many considering the cost and easiness in large-scale implementation. Figure 1 shows the schematic of the double-layer design, in which two different layers are used to scatter solar radiation in different wavelength regimes. As UV light, with its short wavelength, normally has a much smaller penetration depth than visible and near-infrared radiation, it is a natural idea to first scatter the UV radiation and some short-wavelength visible light using a wide-bandgap material as the top layer while the other layer to reflect visible light and near-infrared radiation.

To study the effects of different parameters of the double-layer painted film and achieve the optimal design, numerical simulations were conducted by combining the Mie theory and Monte Carlo simulations. Mie scattering [44] is the main scattering mechanism being considered for cooling paints as the sizes of the particles are normally comparable with the solar radiation wavelength (0.25–3 μm) while the Rayleigh scattering starts to play a role when the particle sizes are much smaller than the light wavelength. The Lorentz-Mie theory models the interactions between the light of a wavelength *λ* and a spherical particle of a diameter *d* and a complex refractive index of *m* + *ki* by a set of governing equations. In this study, we adopt the approach from Bohren [33], where the complex refractive index of the particle is normalized by the index of the matrix. However, the corresponding governing equations of Mie scattering are often formulated for a single particle, and they need to be adjusted for a group of particles when studying paints.

The open-source code by Maetzler [34] is used to calculate the Mie scattering properties by a group of particles [35], [36], including the effective scattering efficiency (*σ*_*T*_), effective absorbing efficiency (*κ*_*T*_) and effective asymmetric factor (*g*_*T*_), as shown in Equations (1)–(3), where c is the number of particle sizes, *d*_*i*_ is the diameter of each particle, and *f*_*i*_ is the volume fraction of each particle. *Q*_sca,*i*_ is the scattering efficiency, *Q*_abs,*I*_ is the absorbing efficiency, and the *g*_*i*_ is the asymmetric factor of a particle with a diameter of *d*_*i*_.

(1) $$\sigma_{T}=\overset{c}{\underset{i=1}{\sum }}\frac{3f_{i}Q_{\text{sca},i}}{2d_{i}}$$

(2) $$\kappa_{T}=\overset{c}{\underset{i=1}{\sum }}\frac{3f_{i}Q_{\text{abs},i}}{2d_{i}}$$

(3) $$g_{T}=\frac{1}{\sigma_{T}}\overset{c}{\underset{i=1}{\sum }}\frac{3f_{i}g_{i}Q_{\text{sca},i}}{2d_{i}}$$

**Figure 2:**
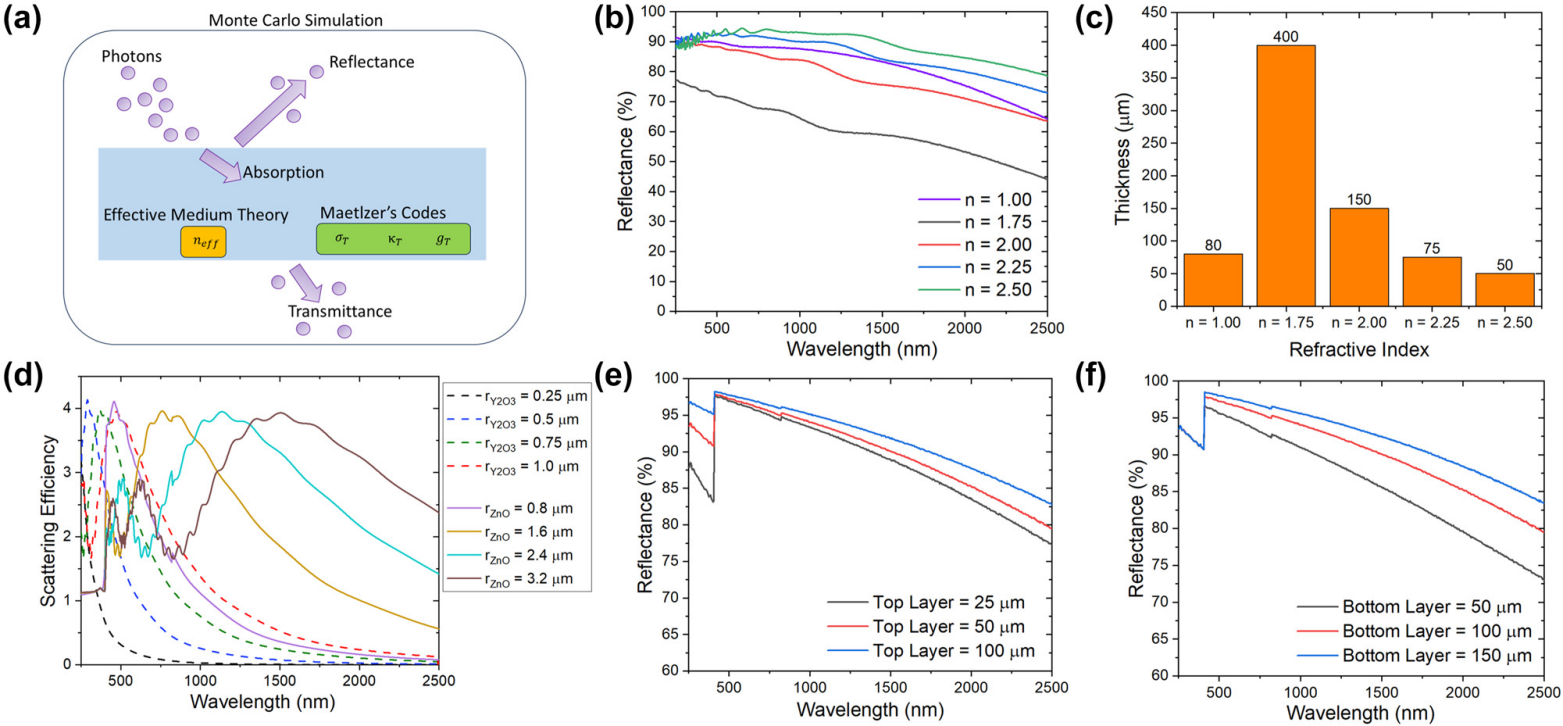
Simulation and calculations. (a) Schematic of simulation process. (b) Reflectances of 50-μm-thick paints with 800-nm fillers of different refractive indexes. (c) Required film thicknesses for fillers with different refractive indexes to achieve a reflectance of 92 %. (d) Scattering efficiency of ZnO and Y_2_O_3_ particles of different sizes. (e) Variation of the spectral reflectance of the paint with respect to the top layer thickness while maintaining the bottom layer thickness of 100 μm. (f) Variation of the spectral reflectance of the paint with respect to the bottom layer thickness while maintaining the top layer thickness of 50 μm.

To investigate the light scattering in the polymer composites with various particles of different sizes, the Monte Carlo simulation method is adopted, which tracks a photon’s propagation in the composite and monitors the different events including light absorption or scattering. These events occur according to the values of scattering and absorption efficiencies from Mie scattering theory [37], [38], and the effective complex refractive index of the composite from effective medium theory [39]. The Monte Carlo simulation was conducted using an open-source code called Monte Carlo for multi-layer media by Wang et al. [40]. 500,000 photons were emitted into the paint film at a selected wavelength value. In total, 451 wavelength values are uniformly sampled from 250 nm to 2500 nm. The statistical results were then used to determine the reflectance, absorptance, and transmittance spectra. Because of the high concentration of the particles, a dependent scattering correction was implemented to improve accuracy [41]. More details can be found in studies by Li et al. and Peoples et al. [29], [36]. The simulation and calculation process are shown in Figure 2a.

A 50 μm thick paint film was first selected for the Monte Carlo simulation, which consists of an acrylic resin matrix with a refractive index of 1.49 and filler particles of different refractive indexes. The volume concentration of the fill material is set at 50 % and the particle size of the material is 800 nm. When the refractive indexes of the filler particles are 1.00, 1.75, 2.00, 2.25, and 2.50, the corresponding reflectance of the film is 87.7 %, 67.3 %, 84.5 %, 90.4 %, and 92.3 %, respectively. Apparently, for the same matrix and filler size, increasing the refractive index contrast between the particles and the matrix can improve the reflectance. For example, a 37 % increase in reflectance can be achieved when changing the refractive index from 1.75 to 2.5. A 30 % increase in reflectance can also be achieved when changing the refractive index from 1.75 to 1.00, showing air pores to be efficient in scattering light (Figure 2b). On the other hand, Figure 2c shows that to achieve the same reflectance, a larger refractive index contrast can lead to much-reduced thickness. For example, to achieve the same reflectance of 92 %, the required film thickness can be reduced by 87.5 % when increasing the refractive index from 1.75 to 2.5. Thus, selecting fillers with a higher refractive index is critical for reducing the thickness of a painted film.

Common materials that have high refractive indexes and are widely used in white paints are titanium dioxide and zinc oxide due to their white appearance. However, these materials have their drawbacks since they also have an intrinsic nature to absorb UV radiation which can prevent effective radiative cooling. This absorption comes from their relatively narrow band gaps of around 3 eV so that the corresponding paints generally show reflectance below 92 % [36]. Thus, we propose to adopt two different filler materials that can target different regions of the solar spectrum based on their refractive indices. Y_2_O_3_ was chosen to be the top layer of the double-layer structure because of its relatively high refractive index of approximately ∼2.0 in the UV regime [42] to achieve strong UV scattering and a wide band gap of 5.5 eV [43] to avoid UV absorption. ZnO with a band gap of around 3.4 eV was chosen for the bottom layer for its high refractive index of ∼2.1 in the visible and NIR regimes, which can lead to strong scattering in visible and NIR light.

The scattering efficiency depends on the intrinsic optical properties of the filler materials as well as the particle size. The scattering efficiencies are shown in Figure 2d. Figure 2d shows that the scattering efficiency peaks in different wavelength regimes for particles of different sizes, indicating that a wide distribution of particle size is beneficial to scatter light throughout the wide spectrum. This is also supported by a study by Peoples [36]. According to the Mie scattering results shown in Figure 2d, Y_2_O_3_ particles of a size between 300 and 800 nm show the highest efficiency in the UV region while ZnO particles of a size of 800–1200 nm show the highest scattering performance in the visible range and NIR regimes. The average scattering efficiency was also calculated by taking the weighted average of the solar spectrum shown in Figure S1. The Y_2_O_3_ particles with sizes ranging from 500 to 800 nm show the highest scattering efficiency in the UV region, while ZnO particles ranging from 800 to 1200 nm have the highest scattering efficiency in the visible regime. Based on these calculations, powders from different vendors were tested to select the optimal sizes of Y_2_O_3_ and ZnO particles. Simulations were further conducted based on the actual distribution of particle sizes. The volume concentrations of particles were determined by Monte Carlo simulations at a set thickness of 50 µm. The volume concentration was varied from 50 % to 65 % as shown in Figure S2. For the Y_2_O_3_ paint, the reflectance increases from 83.1 % to 85.3 % as the concentration increases while for the ZnO paint, the reflectance increases from 82.0 % to 83.8 %. Although the simulations suggest the values of the volume concentration of the paints, the volume concentration of Y_2_O_3_ was set to 55 % and ZnO to 60 % because tests showed that a too-high volume concentration could cause difficulties such as cracking and low adhesion in fabrication.

The optical properties of different double-layer designs were simulated by the Monte Carlo simulations and the results are shown in Figure 2e and f. The purpose is to study the effects of the top and bottom layer thicknesses and to optimize the design while keeping the film no thicker than 200 µm and with a predetermined volume of Y_2_O_3_ and ZnO paint of 55 % and 60 %, respectively. The top layer thicknesses of 25 µm, 50 µm, and 100 µm were chosen for illustration while maintaining a thickness of 100 µm for the bottom layer. From Figure 1e, increasing the top layer thickness mainly improves the UV reflectance while the reflectance in the NIR region is also enhanced. The overall solar reflectance improves from 93.5 % to 95.7 % when the top layer thickness increases from 25 µm to 100 µm. Then, the top layer thickness was set as 50 µm while the bottom layer was set to be 50 µm, 100 µm, and 150 µm. The reflectance increases over the entire visible and NIR spectra when the bottom layer is thicker, as shown in Figure 1f. However, the UV reflectance remains constant, indicating the dominant contribution of the top layer in scattering UV. To achieve both a high solar reflectance and a low overall thickness, a paint structure with a 50-µm Y_2_O_3_ top layer and 100-µm ZnO bottom layer was selected, with a simulated reflectance of ∼95 %.

## 3 Fabrication and characterization

**Figure 3:**
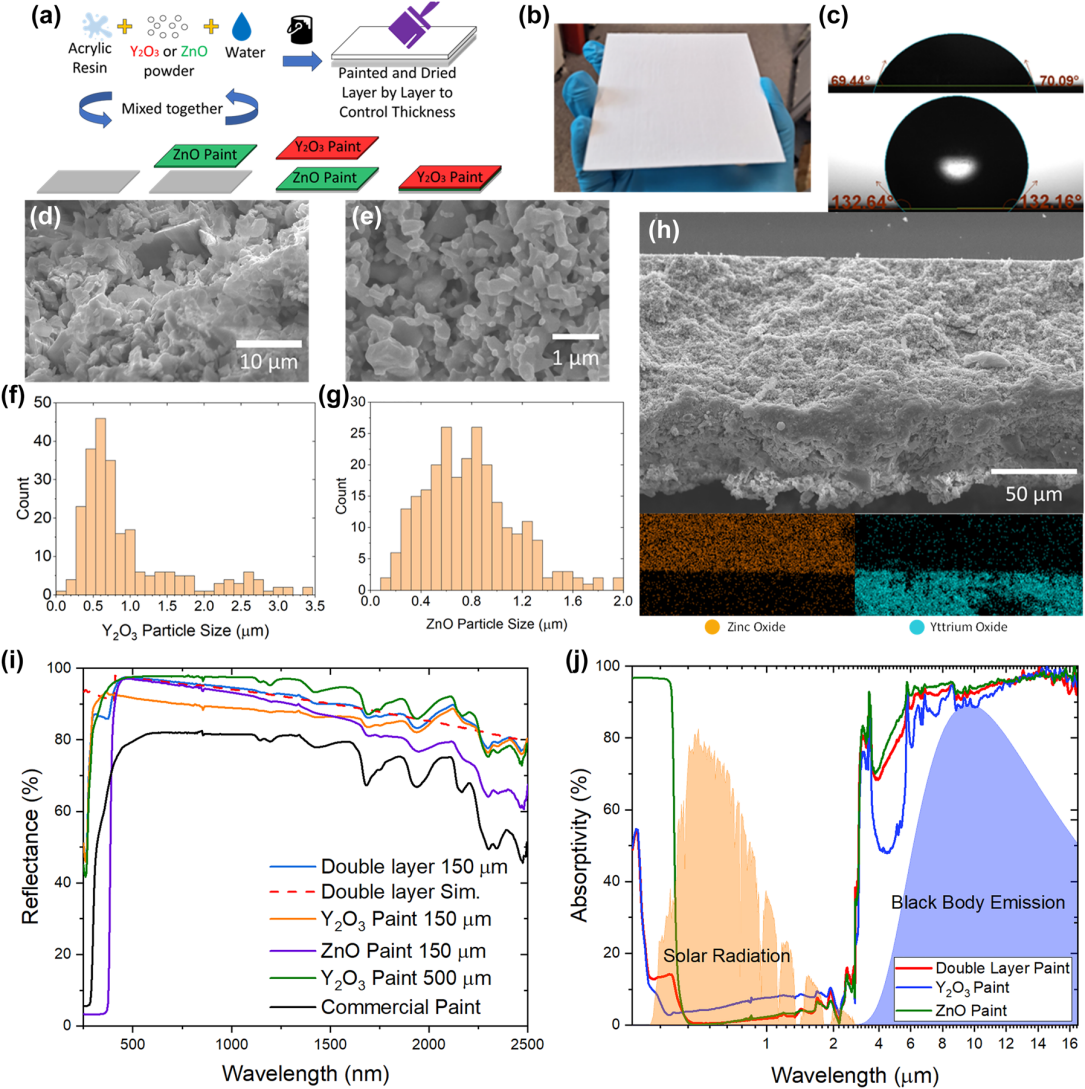
Fabrication and characterization. (a) Fabrication process of double layer paint. (b) Double-layer paint sample on a 10 × 10 cm aluminium substrate. (c) Contact angle of commercial paint (top) and radiative cooling paint (bottom). (d) SEM of Y_2_O_3_ paint. (e) SEM of ZnO paint. (f) Y_2_O_3_ particle size distribution. (g) ZnO particle size distribution. (h) SEM of cross-section double layer paint with EDS of cross-section showing ZnO and Y_2_O_3_. (i) Comparison of different paints. (j) Full spectrum of multilayer paint, Y_2_O_3_ paint, and ZnO paint.

From the above simulations, the optimal average particle size for the Y_2_O_3_ top layer should be in the range of 300–500 nm, and that for the ZnO bottom layer is 800–1000 nm. Y_2_O_3_ and ZnO powders from different vendors were tested for the particle sizes and the powders that were purchased from Hebei Badu Metal Materials Co., Ltd showed the best match for the optimal particle sizes. Figure 3a shows the fabrication process of the double-layer coating. First, acrylic resin (Xuzhou Lvyuan Chemical Co., Ltd) and the fillers (Y_2_O_3_ or ZnO particles in this work) with simulated volume concentration (55 % for Y_2_O_3_ and 60 % for ZnO in this work) are mixed. Water with the same amount as the resin is added to the mixture and the solution is stirred using a homogenizer. The bottom ZnO layer and the top Y_2_O_3_ layer were applied in sequence onto a 1-mm-thick aluminum substrate by multiple times coating with a paintbrush. The thickness was measured by a micrometer after the coating of each layer. Around 10-μm thickness was gained for each coating, which benefited from the low viscosity of the paint. The total thickness of the film was controlled accurately by the times of painting. The thickness of each coat was calibrated using some dummy samples to determine the number of coatings for the two different layers. The coated samples were then left on a hotplate at 100 °C for at least 15 min to completely dry out. Figure 3b shows a fabricated sample on a 10 × 10 cm aluminium substrate. The coating shows hydrophobicity and the contact angle is around 130°, as shown in Figure 3c.

To understand the painted film structure, the cross-sections of the Y_2_O_3_ and ZnO paints and the multilayer paint were characterized by a scanning electron microscope (SEM) and illustrated in Figure 3d, e, and h. Figure 3f shows the size distribution of Y_2_O_3_ particles, which mainly fall in the range from 200 to 1000 nm and peak at 510 nm. The size distribution in Figure 3g indicates that the majority of ZnO particles have a size between 500 and 1000 nm with the peak distribution near 800 nm. The SEM images of the Y_2_O_3_ and ZnO layers also reveal that the layers are porous structures while the top layer is denser, which may lead to some discrepancies between the simulation and experimental results. From the SEM of the cross-section of the multilayer paint, the thickness is approximately 141 μm with a clear separation of the two layers. Figure 3h shows the energy dispersive X-ray spectroscopy (EDS) results, which verify the distinct separation of the ZnO and Y_2_O_3_ layers. Figure 3i compares the reflectance spectra of different paints from 250 to 2500 nm, which were measured with a UV-visible-near-infrared spectrometer (Perkin Elmer Lambda 1050+). The 150-µm-thick double-layer paint shows a solar reflectance of 95.4 % which is much higher than that of 79.1 % of a typical commercial heat-reflecting paint based on TiO_2_ (Beijing Zhisheng Weihua Technology Co., Ltd.). The simulated reflectance spectra are similar to the measured results of the double-layered paint, verifying the reliability of the simulation results. The double-layered paint was also compared with Y_2_O_3_ and ZnO monolayer paints with the same thickness of 150 µm. The Y_2_O_3_ and ZnO monolayer paints show reflectances of 89.8 % and 90.8 % respectively, both of which are lower than that of the double-layer structure (95.4 %). This indicates the synergetic effects of the Y_2_O_3_ and ZnO layers in the double-layer design. It is also noted that the reflectance of the 150-µm-thick double-layer paint is very close to that of a 500-µm-thick painted film of Y_2_O_3_ (96.3 %), despite its much smaller thickness.

To clarify the relative contribution of each layer and their interaction, the spectra of a separate 100-μm ZnO layer and 50-μm Y_2_O_3_ layer are also measured for comparison in Figure 3j. The 100-μm ZnO layer shows a solar reflectance of 89.2 % while that of the 50-μm Y_2_O_3_ layer is 82.7 %. It is noted that the ZnO layer has a very high reflectance in the visible light and near-infrared regimes, but it shows high absorption in the UV regime (<400 nm), which limits its overall solar reflectance. In contrast, the 50-μm Y_2_O_3_ layer demonstrates a high solar reflectance below 400 nm, although the reflectance above 400 nm is generally less than that of the ZnO layer. The complementary spectra of these two layers enable the double-layer design, i.e., the UV light can be mainly reflected by the Y_2_O_3_ layer while the light in the visible and near-infrared regimes can be mainly reflected by the ZnO layer. The measured thermal emissivities of the 50-μm Y_2_O_3_ paint, 100-μm ZnO paint, and 150-μm double-layer paint are 0.92, 0.95, and 0.93, respectively, in the atmospheric window. From Figure 3g, it can be seen that the emissivity of the double-layer paint seems to be mainly contributed by the top Y_2_O_3_ paint layer as its thermal emission spectrum is almost the same as that of 50-μm Y_2_O_3_ paint but quite different from that of the separate ZnO layer. Further studies are shown in the higher thickness of the Y_2_O_3_ and ZnO paint in Figure S4a. In addition, another type of double-layer paint was fabricated with the same method but with TiO_2_ as the bottom layer and the test also shows improved performance (Figure S4b) similar to those of Y_2_O_3_–ZnO paints, implying that this design can adopt different white high-refractive-index fillers for the bottom layer.

## 4 Field tests

**Figure 4:**
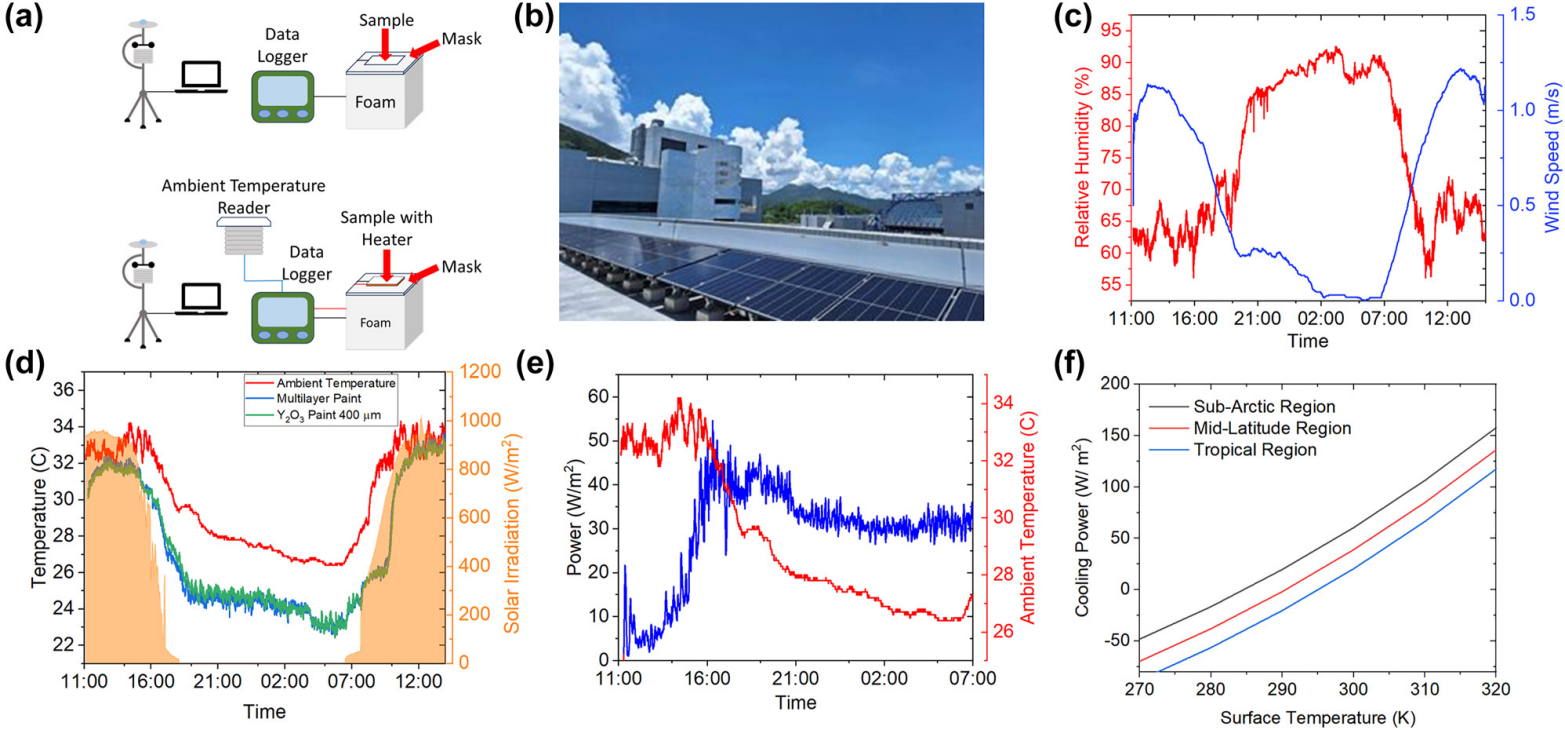
Field testing setup and results. (a) Field test setups for cooling temperature (top) and cooling power (bottom). (b) Sky condition in HKUST. (c) Relative humidity and wind speed during the field test. (d) Temperature variation during the field test. (e) Cooling power versus ambient temperature throughout the field test. (f) Calculated daytime cooling power at various surface temperature in different climate regions.

To evaluate the cooling performance of the cooling paint, field tests were conducted on sunny summer days on the roof of a building in HKUST (Figure 4b), Hong Kong during 2–3 August 2023. The samples were painted using a brush to the required thickness after drying on 10 × 10 cm aluminium plates. The painted samples were then directly placed on a white polystyrene foam cube (30 × 30 × 30 cm) with a low thermal conductivity (∼0.03 W/mK) to prevent heat transfer from the edge and backside, as shown in Figure 4a. Note that we adopted the “roof cooling mode”, i.e., no convention cover was used during the test to show its performance in practical applications. To further thermally isolate the samples, a commercial radiative cooling film with 93 % solar reflectance was placed around the samples to reduce the solar absorption by the adjacent foam surface. The plate temperature was measured continuously by a T-type thermocouple attached to the backside of the sample. A calibrated commercial weather station was used to record the real-time weather data, including solar irradiation, ambient temperature, relative humidity, and wind speed. The data collected from the weather station are in good agreement with the data from the HKUST Air Quality Research Supersite Facility shown in Figure S5d–f.

The weather station showed a peak solar irradiation of ∼950 W/m^2^ at noon and the highest air temperature of 34 °C appeared at around 3:00 pm. The average relative humidity was 60 % during the daytime and 90 % at nighttime. Figure 4c shows the real-time temperatures of the ambient air, 150-µm double-layer paint, and 500 µm Y_2_O_3_ paint. Both paints were able to maintain a sub-ambient temperature throughout the field-testing period. Both paints achieved a sub-ambient cooling temperature of ∼2 °C when the ambient air temperature was the highest and ∼4 °C during the night. This test shows that the 150-μm double-layer paint has comparable performance with the 500-μm Y_2_O_3_ paint even at a much smaller thickness.

Other than the cooling temperature, the cooling power of the sample was also measured by the setup shown in Figure 4a on the same day of the field test. An electric heater was attached to the backside of the plate, of which the temperature was monitored by a thermocouple. Another shaded thermocouple was used to measure the ambient air temperature. The measured temperature difference was input to a proportional–integral–derivative (PID) controller to control the heating power of the heater to match the sample’s temperature to the ambient temperature. The cooling power was then obtained from the heating power of the heater according to the first law of thermodynamics, which is displayed in Figure 4e. The highest cooling power of ∼50 W/m^2^ was achieved at night while around 10 W/m^2^ when the air temperature was highest.

The cooling performance of a radiative cooler will vary under different climates, depending on solar irradiation, humidity, wind speed, and cloud coverage. To assess the cooling capability of the double-layer paint in different climates, we calculated the cooling power and cooling temperature at 300 K during daytime in three different climates using Equation (4). The atmospheric conditions are set in the Sub-arctic, mid-latitude, and tropical areas provided by MODTRAN during the summer. The non-radiative heat loss (*P*_nrad_) was assumed to be zero. The solar radiation (*P*_sun_) is also set to be 1000 W/m^2^ in the daytime with the ambient temperature set at 300 K. The results are shown in Figure 4f. The figure shows that different climates affect the cooling potential of the radiative cooler performance. The performance is the lowest in the Tropical climate but highest in the sub-artic climates. During the daytime, the equilibrium cooling powers are 60.5 W/m^2^, 39.0 W/m^2^, and 20.5 W/m^2^ during the nighttime. This shows the different climates can have approximately 20 W/m^2^ difference between each climate. This is due to the relative humidity of each climate affecting the transparency of the atmospheric window [44]. Sub-arctic regions have the lowest relative humidity and tropical regions have the highest while the mid latitude is between the two.

(4) $$P_{\text{cooling}}=P_{\text{rad}}-P_{\text{sun}}-P_{\text{atm}}-P_{\text{nrad}}$$

## 5 Durability test

**Figure 5:**
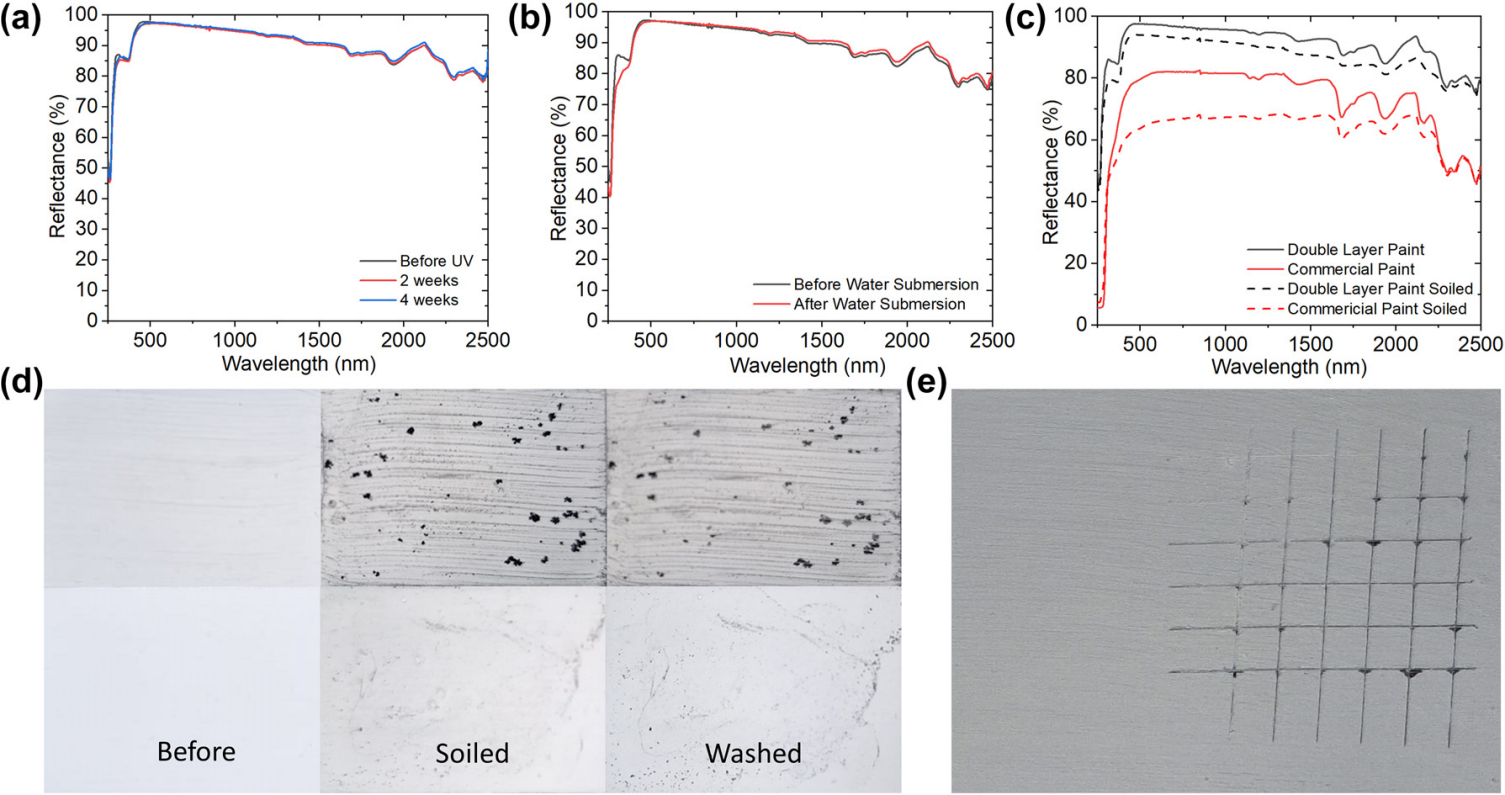
Weather tests. (a) Comparison of reflectance of different durations of UV light. (b) Comparison of reflectance of before and after water submersion. (c) Comparison of reflectance of double layer paint and commercial paint before and after soiling test. (d) Comparison of soiling test of commercial paint and radiative cooling paint. (e) Adhesion test.

Most organic materials including paints tend to degrade under UV irradiation or moisture, which may limit their lifetime and cooling performance in long-term applications [45]. To test the UV resilience of the paint, an accelerated UV durability test was conducted with a 40-W UV test chamber with a UV radiation intensity equivalent to that in the standard 1-sun irradiation. The optical properties of the painted samples before and after the UV test are shown in Figure 5a, illustrating negligible change in the reflectance spectrum. The total solar reflectance before the UV test was 94.8 % while that after the UV test was 94.9 % after 16 weeks of daytime UV exposure. The variation may be due to the uncertainties in the spectrometer. During practical applications, moisture may cause the degradation of the cooling performance of the coating, especially when water is absorbed or trapped in the coating structure. To test the resistance to water, a paint sample was submerged in water for five days and then dried. Its optical properties were measured before and after the test, as shown in Figure 5b. The total solar reflectance before the test is 94.2 % and 94.4 % after the test, showing good water resistance.

The soiling test was conducted by mixing carbon black powder with water at a concentration of 0.03 g of powder per 1 L of water, as performed by a study by Song [27]. The mixture was then dropped on the painted samples and heated. The samples were then washed, and the reflectance spectra were compared. Figure 5d shows the result of the soiling testing of the commercial white paint and the radiative cooling paint. Due to the higher hydrophobicity of the radiative cooling paint, the carbon black dust can be easily washed away while the commercial paint had large spots of carbon black. Their reflectance spectra were also measured and shown in Figure 5c. The solar reflectance of the commercial paint decreased from 79.1 % to 65.1 % after the soiling test while the double-layer paint showed a higher resistance, only showing a 3.5 % reduction in the solar reflectance. To test the stability over time, a sample of Y_2_O_3_ painted film of 500 μm that was exposed to air at room temperature for more than a half year was tested multiple times with the UV–vis spectrometer. It was observed that there was negligible variation in the reflectance spectra of the film (Figure S6), which showed a solar reflectance of 96.1 % at the beginning and 96.0 % after half a year.

A good paint must be able to adhere to a surface well and will not peel off easily. The standard cross-cut tape test according to the ISO2409-2020 standard was conducted, in which a tool was used to scratch grids on the painted film and then a standard tape was then placed on the grid and peeled off the film. Figure 5e shows there was little to no paint peeling off after the test, indicating that the paint has a high grade of adhesion ISO1 to the surface.

## 6 Conclusions

We have reported a double-layer design to produce a thin radiative cooling painted film with a high solar reflectance by utilizing a multilayer structure painted film. The structure and the performance of the painted film were studied by numerical simulations and experiments. The 150-um painted film was able to have a 95.2 % reflectance and 0.93 emissivity which is the same as a Y_2_O_3_ paint of 500 um thickness, showing significant thickness reduction by the design. From the field test, the painted film was able to maintain a cooling temperature of 2 °C at noon time and 4 °C at nighttime. The cooling power was also recorded through field testing, resulting in a peak cooling power of 50 W/m^2^ at nighttime and 10 W/m^2^ during the daytime. The painted film also illustrates good hydrophobicity, high adhesion strength, and excellent resistance to water and UV light attacks, showing promise for large-scale applications. Y_2_O_3_ may be more costly compared to other types of powder but other cheaper materials can also be used for the top layer if the materials with proper properties scatter UV light efficiently.

## Supplementary Material

Supplementary Material Details

Supplementary Material Details
