# Supplementary material for: Aqueous double-layer paint of low thickness for sub-ambient radiative cooling
Source: Nanophotonics. 2024 Jan 18;13(5):659–68. doi: 10.1515/nanoph-2023-0664 (PMC11501399; doi:10.1515/nanoph-2023-0664)
Supplement: Supplementary file 2 — Supplementary Material Details [file j_nanoph-2023-0664_suppl_002.docx]

Supplementary Materials

Aqueous double-layer paint of low thickness for sub-ambient radiative cooling

(b)

(a)


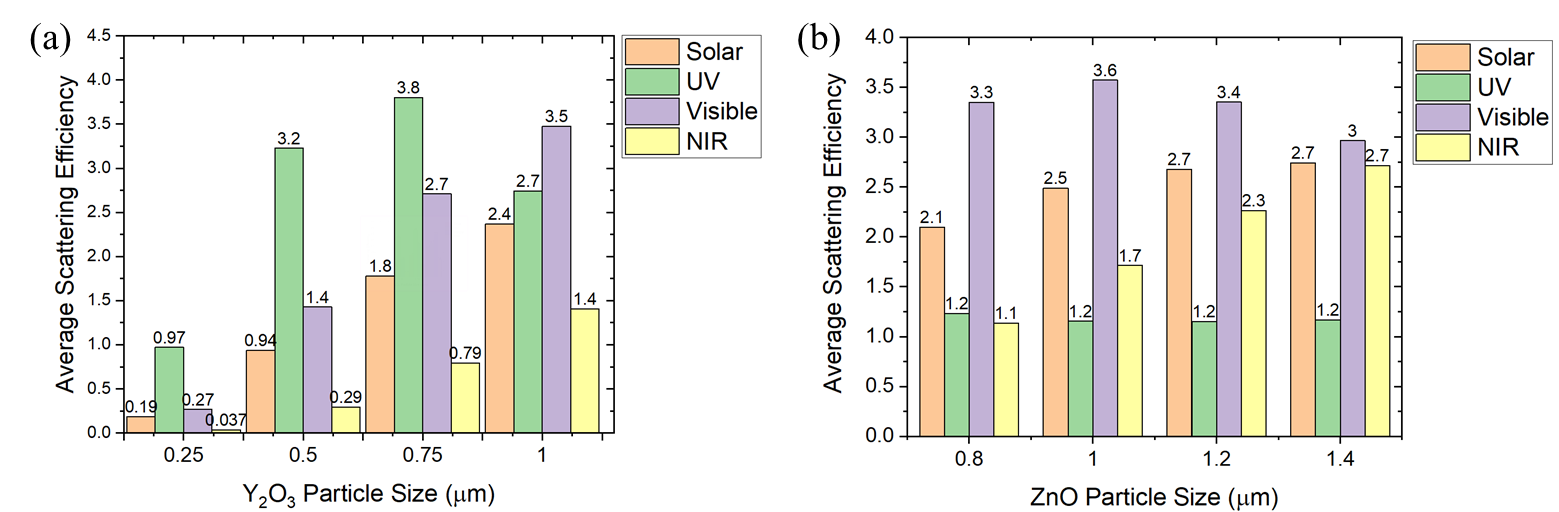


Figure S1:

Scattering Efficiency of (a) Y_2_O_3_ and (b) ZnO particles in different solar spectrum regions.


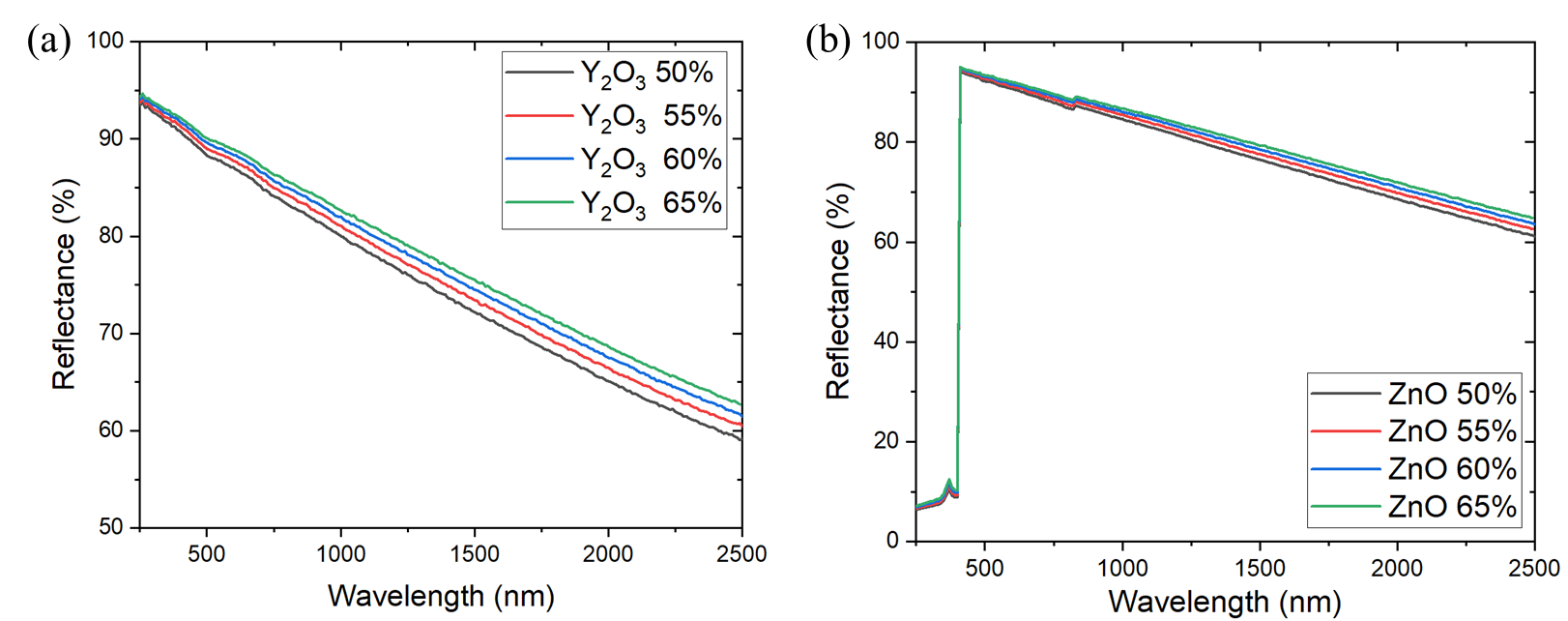


Figure S2:

Simulation results of Reflectance of different volume concentrations of (a) Y_2_O_3_ and (b) ZnO paint.


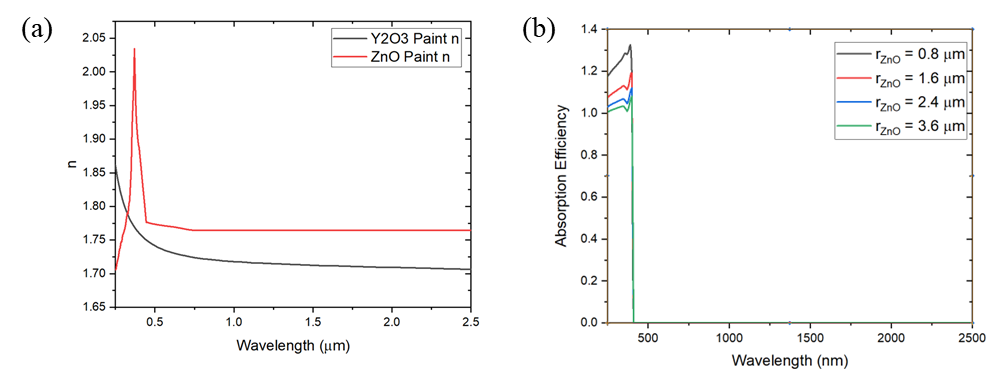


Figure S3:

(a) Effective Refractive Index n of Y_2_O_3_ and ZnO paint. (b) Absorption Efficiency of ZnO particles


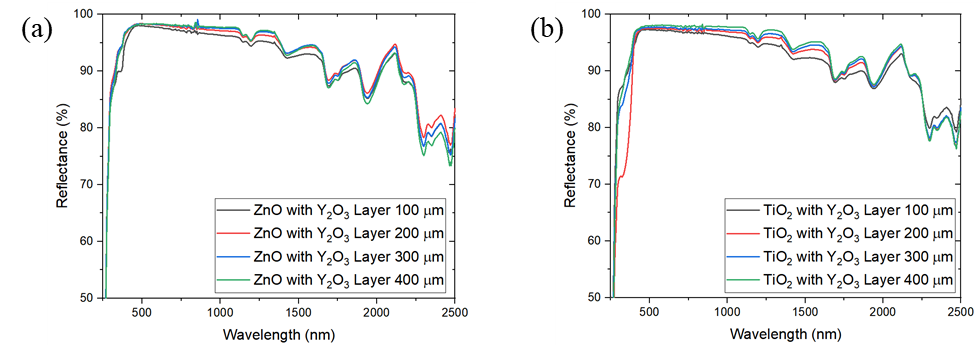


Figure S4:

(a) Reflectance of double-layer Y_2_O_3_-ZnO paints with the top Y_2_O_3_ layer thickness ranging from 100 to 400 microns. (b) The Reflectance spectra of double-layer Y_2_O_3_-TiO_2_ paint with the top Y_2_O_3_ layer thickness ranging from 100 to 400 microns.


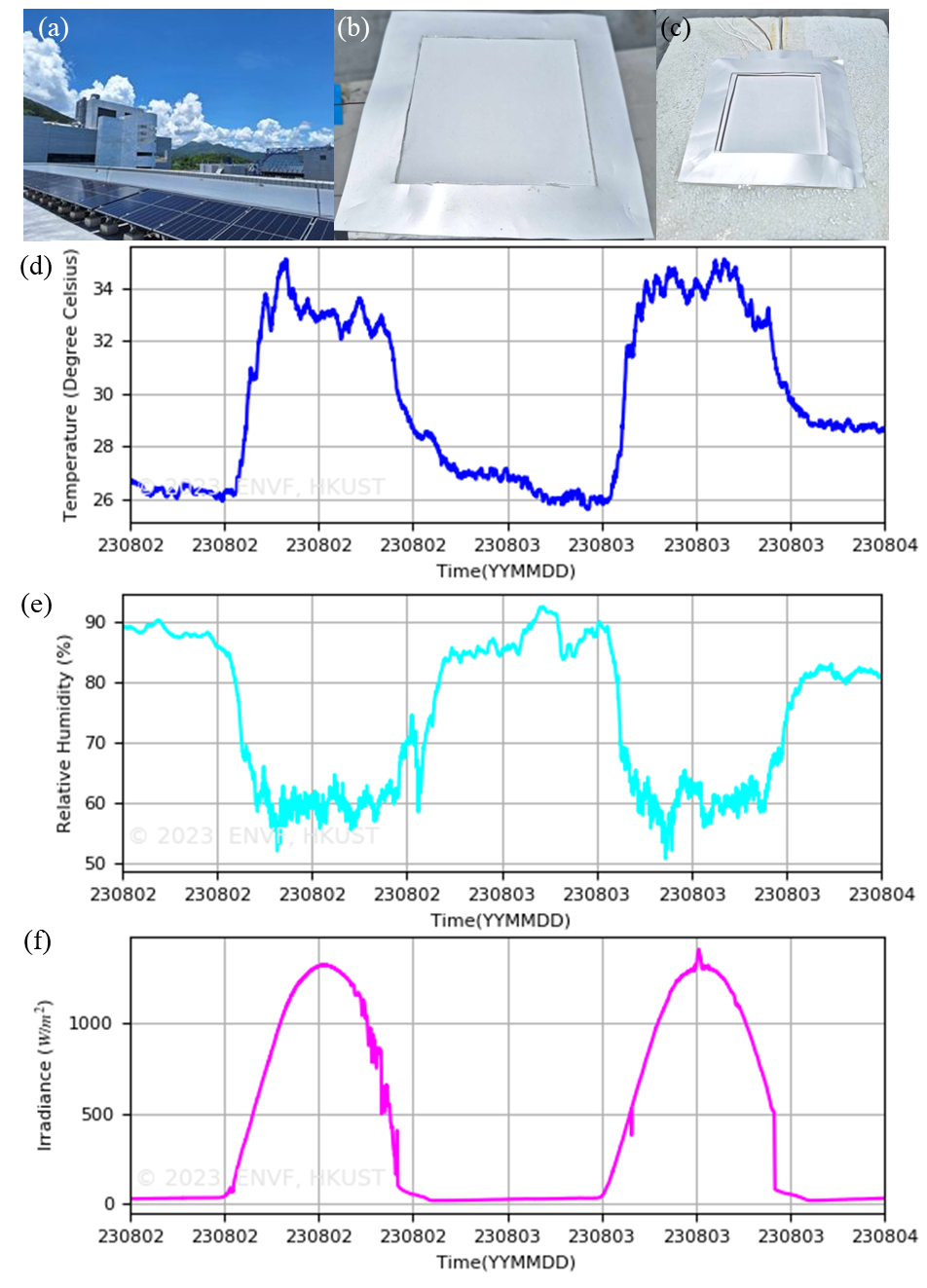


Figure S5:

(a) Picture of Sky during field testing. (b) Temperature measurement in the field testing. (c) Cooling power measurement in the field testing. (d) Temperature, (e) relative humidity, and (f) solar irradiation data from HKUST Supersite Research Facility


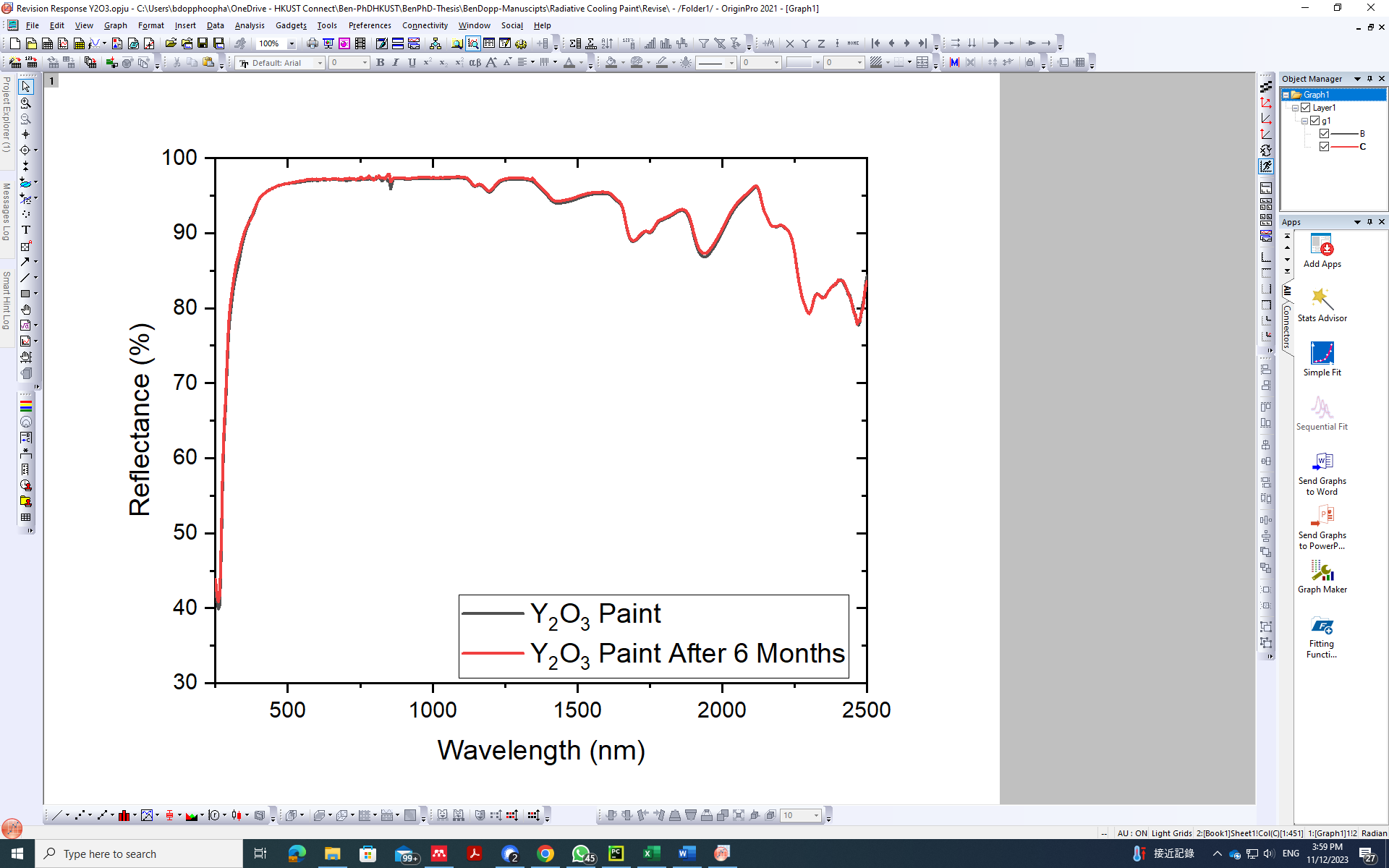


Figure S6:

Reflectance of Y_2_O_3_ 500 microns paint before and after 6 months of exposure to room temperature environment

**Equipment Used in Study**

(b)

**1 Optical and Emissivity Performance Measurement**

1.1 UV-Vis Spectrometer

When studying the performance of a radiative cooling coating, the optical properties need to be measured. The instrument that was used for our study is the UV-Vis Spectrometer. This spectrometer uses a light source to shine on a sample to measure transmittance and reflectance. The model that was used is Perkin Elmer Lambda 1050+. The machine uses two monochromators that cover wavelengths of 175 to 3300 nm. The wavelength range that was set was 250 to 2500 nm. Because of this, the instrument was used to study the performance of the coating in the solar spectrum.

1.2 FTIR (Fourier Transform Infrared) Spectrometer

Another parameter that needs to be studied for radiative cooling is emissivity. The emissivity of a coating needs to be measured at long wavelengths beyond 2500 nm. To measure this, a Fourier Transform Infrared Spectrometer was used to measure the emissivity of the coating. The model of the instrument that was used is the Fourier transform infrared spectroscopy (FTIR, Vertex 70, 275 Bruker) together with a gold integrating sphere (PIKE Technologies). The reflectance and transmittance were measured, and the absorption can be calculated to find the emissivity.

**2 SEM Characterization**

The particle size of the materials and cross-section of the paint were characterized by scanning electron microscopy (SEM, JSM-6700F). It is a high-resolution and easy-to-operate scanning electron microscope, which employs a field-emission gun for the electron source and state-of-the-art computer technology for the image-display system.

The particle size of ZnO and Y_2_O_3_ powders were measured and used for data for the particle used in the Monte Carlo simulation. SEM Image was also used to study the cross-section of the paints.

**3 UV Stability Test**

Acrylic resin is an organic polymer that is commonly used for mixing and making paint. In most cases, organic materials are prone to UV degradation due to the high energy of UV radiation breaking polymer chains. This causes free radicals release and reduces the molecular weight leading to deterioration of mechanical properties and intended optical properties. This is an important aspect to test since radiative cooling paints must be applied outside facing UV radiation within the solar spectrum. Acrylic resin is one of many polymers that are inherently UV resistant which is why it is a popular resin to use in the painting industry.

To test the UV resistance of the developed radiative cooling paint, the painted film will be placed in a UV Chamber that has 40 W and a peak of 340 nm wavelength to simulate an accelerated condition of UV exposure. The set time for exposure in the UV chamber is 2 weeks. After 2 weeks in the chamber, the sample optical properties in the solar spectrum were tested by measurements from the spectrometer. The measurements were then compared with the measurements before the sample was placed in the chamber. If the sample showed a lot of variation in optical properties, UV degradation has occurred.

**4 Contact Angle Measurement**

The water contact angle measure serves as a way to study the interaction between water and the painted film. This is done by using the Biolin Theta Contact Angle Meter by placing a water droplet on the painted film and measuring the contact angle between the droplet and film.
